# Unveiling the contribution of the reproductive system of individual *Caenorhabditis elegans* on oxygen consumption by single-point scanning electrochemical microscopy measurements

**DOI:** 10.1016/j.aca.2020.12.030

**Published:** 2021-02-15

**Authors:** Carla S. Santos, Felipe Macedo, Alicia J. Kowaltowski, Mauro Bertotti, Patrick R. Unwin, Fernanda Marques da Cunha, Gabriel N. Meloni

**Affiliations:** a Departamento de Química Fundamental, Av. Professor Lineu Prestes, 748, 05508-000, São Paulo, SP, Brazil; b Departamento de Bioquímica, Instituto de Química, Universidade de São Paulo, Av. Professor Lineu Prestes, 748, 05508-000, São Paulo, SP, Brazil; c Departamento de Bioquímica, Escola Paulista de Medicina, Universidade Federal de São Paulo, Rua três de Maio, 100, 04044-020, São Paulo, Brazil; d Department of Chemistry, University of Warwick, Coventry, CV4 7AL, United Kingdom; e Bio-Electrical Engineering Innovation Hub, University of Warwick, Coventry, CV4 7AL, United Kingdom

**Keywords:** Respiration, OCR, SECM, C. elegans, Ultramicroelectrode, FEM

## Abstract

Metabolic analysis in animals is usually either evaluated as whole-body measurements or in isolated tissue samples. To reveal tissue specificities *in vivo*, this study uses scanning electrochemical microscopy (SECM) to provide localized oxygen consumption rates (OCRs) in different regions of single adult *Caenorhabditis elegans* individuals. This is achieved by measuring the oxygen reduction current at the SECM tip electrode and using a finite element method model of the experiment that defines oxygen concentration and flux at the surface of the organism. SECM mapping measurements uncover a marked heterogeneity of OCR along the worm, with high respiration rates at the reproductive system region. To enable sensitive and quantitative measurements, a self-referencing approach is adopted, whereby the oxygen reduction current at the SECM tip is measured at a selected point on the worm and in bulk solution (calibration). Using genetic and pharmacological approaches, our SECM measurements indicate that viable eggs in the reproductive system are the main contributors in the total oxygen consumption of adult *Caenorhabditis elegans*. The finding that large regional differences in OCR exist within the animal provides a new understanding of oxygen consumption and metabolic measurements, paving the way for tissue-specific metabolic analyses and toxicity evaluation within single organisms.

## Highlights

• *In vivo*, tissue-specific, oxygen consumption rate over a single worm is measured using scanning electrochemical microscopy.
• Respiration along the body of a *Caenorhabditis elegans* is found to be heterogenous.
• The worm reproductive system respiration is 3-fold that of the head, alluding to the high energetic cost of reproduction.
• Embryonating eggs are the biggest contributors to the reproductive system high respiration rate.
• Smart SECM measurement protocols *in tandem* with numerical simulations enabled high-throughput quantitative analyzes.

## Graphical abstract

**Figure:**
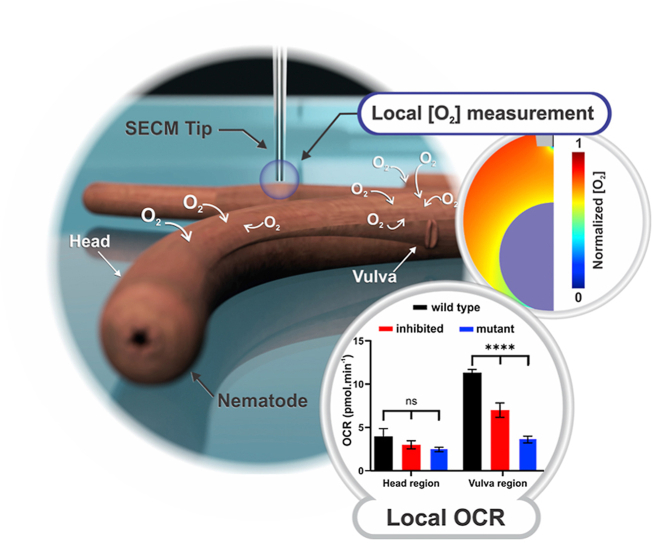

## 1 Introduction

The importance of measuring oxygen consumption rates (OCRs) in living organisms and tissues is clear: as a proxy for metabolic activity, OCRs measurements have elucidated metabolic processes in different systems and enabled a global understanding of metabolic activity at the cellular level [[1], [2], [3], [4], [5]]. Currently, OCRs are assessed at the organism level by measuring bulk oxygen consumption of whole animals or groups of cells. Refined genetic toolkits, allowing gene manipulation in a temporal- and tissue-specific manner, are already available for many organisms*,* [6,7] but the visual mapping of functional parameters such as OCR has not achieved the same level of specificity. In other words, there is a lack of methodology to assess tissue-specific OCRs of whole animals *in vivo*. Developing this capability would allow investigation of metabolic specificities in different animal regions, so as to unveil complex metabolic characteristics of a living animal.

Bulk OCR measurements with a large number of pooled cells, organisms or tissues are more challenging when the organism is small, as in the case of the nematode *Caenorhabditis elegans*. Evaluating OCRs in *C. elegans,* for example, requires 10–1000 worms, depending on the equipment used [8]. Bulk measurements also mask differences in OCRs at the individual organism level, as well as regional body specificities within an organism. Microfluidics technology was recently applied to measure respiration in single worms, in an attempt to circumvent the limitations of bulk measurements [9,10]. However, this technique does not resolve the different OCRs along different parts of the animal body. Information on OCR along the body of a worm would add a new dimension of information, since *C. elegans* (and other organisms) are comprised of different tissues, organized in specific anatomical regions.

Scanning electrochemical microscopy (SECM) is an attractive technique for localized measurements [[11], [12], [13], [14], [15]], and has been used to investigate enzymatic and cellular activity, respiration activity in bovine embryonic cells, tumors, and bacteria, and oxidative imbalance effects on cells [[16], [17], [18], [19], [20], [21], [22], [23], [24], [25]], among other biological applications [26,27]. We have also shown that SECM images can be employed as a tool to qualitatively map oxygen consumption in the vicinity of single human cells, revealing heterogeneous mitochondrial respiratory activity within a population of cells [28]. SECM approach curves and single-point measurements have also been used to quantitatively measure OCR at physically isolated cells [25], and at times using special supports (single-cell conical wells) [21], making those measurements similar to bulk measurements at populations but with single cell resolution. Moreover, these single cells OCR measurements do not consider the interaction between the SECM sensing tip and sample, which can make the measurements unreliable due to known SECM-induced oxygen transfer [29,30]. To access reliable OCR information by SECM, it is necessary to consider the interactions between the SECM probe and the substrate, which cannot be gained by simple numerical approximations [25]. Similar to the microfluid development for single worm OCR measurements [9,10], these approaches represent advances compared to bulk measurements but are still blind to heterogeneities within the sample [21,25]. Although assuming homogenous respiration along the sample might be a reasonable approximation for a single cell, it is certainly not the case for a complex organism, such as the nematode *C. elegans* with around 1000 cells (adult worm) forming different tissues.

Here, we employed SECM imaging to investigate, *in vivo*, local OCR along single adult *C. elegans* worms. Our studies reveal previously unseen hot-spots of respiration along the worm’s body, indicating that the reproductive system has a significant contribution to the overall OCR of adults *C. elegans*. Using this information, we developed a high-throughput, single-point, self-referencing measurement protocol to probe quantitatively the local OCR over the head and middle body regions of a population of worms and, with the aid of finite element method (FEM) simulations, calculated local OCR values. Moreover, we employed pharmacological and genetic approaches to selectively modulate the development of the reproduction system in the nematode, allowing us to gain a better understanding of the contributors of *C. elegans* oxygen consumption. We demonstrate unequivocally that the reproductive system plays the largest role in overall OCR, alluding to a large metabolic reproductive cost [31].

## 2 Experimental section

### 2.1 Chemicals

All chemicals were used as received: phosphate buffer saline (Sigma Aldrich), sulfuric acid (H_2_SO_4_, CAS 7664-93-9, Merck), 2,3-butanedione-2-monoxime (CAS 57-71-6, Sigma Aldrich), 5-fluorouracil (CAS 51-21-8, Sigma Aldrich), *N*,*N*′*-*dicyclohexylcarbodiimide (CAS 538-75-0, Sigma-Aldrich) and dimethyl sulfoxide (CAS 67-68-6, Merck). The solutions were prepared using Nanopure Infinity (Barnstead, USA) purified water.

### 2.2 *C. elegans* strains, culture conditions and sample preparation

Experiments were performed with hermaphrodite *C. elegans* N2 Bristol and *glp-1(e2*144ts*)* (herein referred to as only *glp-1*) strains, as wild-type and germline-less (sterile) animals, respectively. The strains were provided by the Caenorhabditis Genetics Center, which is funded by the NIH Office of Research Infrastructure Programs (P40 OD010440). Worms were cultivated according to standard techniques [32] in Nematode Growth Medium (NGM) plates seeded with *E. coli* OP50-1 and supplemented with 100 μg mL^−1^ streptomycin. For experiments, wild-type worms in the larvae stage L1 were transferred by picking to new NGM plates, and grown at 20 °C until early adulthood, when worms were harvested and used for SECM measurements. For the experiments performed in the presence of 5-fluorouracil (5-FU), animals were prepared in the same manner, but grown to early adulthood in NGM containing 15 μM of 5-FU. Similarly, *glp-1* L1 larvae were transferred by picking to new NGM plates, and grown at 25 °C until early adulthood. Optical images of an example of each condition are shown in the Supplementary Material (Figure S1). For the SECM measurements, worms in the early adult phase were transferred from culture by picking to a Petri dish (TC dish 35, Sarstedt, Germany) containing M9 buffer solution (22 mmol L^−1^ KH_2_PO_4_, 42 mmol L^−1^ Na_2_HPO_4_, 86 mmol L^−1^ NaCl, 1 mmol L^−1^ MgSO_4_) and the anesthetic 2,3- butanedione monoxime (0.3 mol L^−1^), which induces a flaccid paralysis by relaxing the muscles through inhibition of myosin ATPase activity, therefore not increasing ATP synthesis or mitochondrial oxygen consumption [33]. Worms were anaesthetized for approximately 1 h before the beginning of the assay to avoid any movement during the electrochemical measurements.

### 2.3 Electrochemical measurements

All electrochemical measurements were performed using an Autolab PGSTAT128 bipotentiostat/galvanostat (Metrohm Autolab, Netherlands) and a SECM from Sensolytics (Germany), mounted atop an inverted optical microscope (Axio Vert. A1, Zeiss, Germany). The equipment was enclosed by a faraday cage to reduce electrical noise, and supported by an active optical table (TMC, USA) to minimize vibrational noise. Herein, SECM utilized platinum ultramicroelectrodes (UMEs) as mobile tips that enabled spatially-resolved electrochemical measurements [34]. The UMEs were prepared and platinized as previously described [28]. Details of UME preparation, characterization and the platinization processes are given in Section S-2, Supplementary Material. For both imaging and single-point measurements, oxygen was detected by recording cyclic voltammograms (potential sweep: −0.1 to −0.4 V *vs* Ag/AgCl/sat. KCl, sweep rate: 0.1 V s^−1^) at the UME to promote the oxygen reduction reaction (ORR) with the resulting diffusion-limited cathodic current proportional to local oxygen concentration (*vide infra*)*.* By recording rapid cyclic voltammograms (Figure S2c), oxygen was only consumed for a brief period (seconds), and in a small volume as the steady-state concentration profile extends as a hemisphere, approximately 10 radii from the center of the UME [[35], [36], [37]].

#### 2.3.1 SECM oxygen concentration map over a single wild-type *C. elegans* worm

OCR along the body of a nematode was investigated by SECM imaging. The SECM working distance (vertical distance between the UME and the Petri dish surface) was set by recording approach curves towards the Petri dish in the vicinity of a single *C. elegans* (but not over it)*,* using the diffusion-limited ORR current at the UME (hindered diffusion) [34]. Because of the small UME size, the substrate impedes diffusion of O_2_ to the UME only when it is within several μm of the surface, where the current decreases sharply with decreasing electrode/surface separation [37]. This signal thus provided an accurate reference of the surface position for the SECM maps. A representative approach curve can be seen in Section S-3, Figure S3, of the Supplementary Material. With this position defined, the UME position was adjusted so that the end of the electrode was at a distance of 100 μm from the Petri dish bottom (working distance) and translated in the *x-y* plane (parallel to the substrate) at a constant height, stopping at every image pixel where, after a set waiting time (10s of milliseconds), a voltammogram was recorded. The pixel size in the SECM maps was chosen to allow the entire animal length to be scanned in a reasonable time, minimizing stress to the nematode. To correct for substrate tilt, approach curves were recorded at three distinct *x-y* positions along the Petri dish surface, and a matching plane with the final *z* coordinates from all approaches was calculated and used to translate the UME over the substrate at the set constant height. SECM maps were constructed by plotting the steady-state current of the recorded voltammograms at each image pixel. To facilitate comparison between the activity of distinct regions within an electrochemical map, currents were normalized by the largest limiting current obtained at the start of the scan, equivalent to bulk and without the influence of electrode fouling (*vide infra*).

#### 2.3.2 Measuring local OCR by single-point SECM measurements

**Fig. 1:**
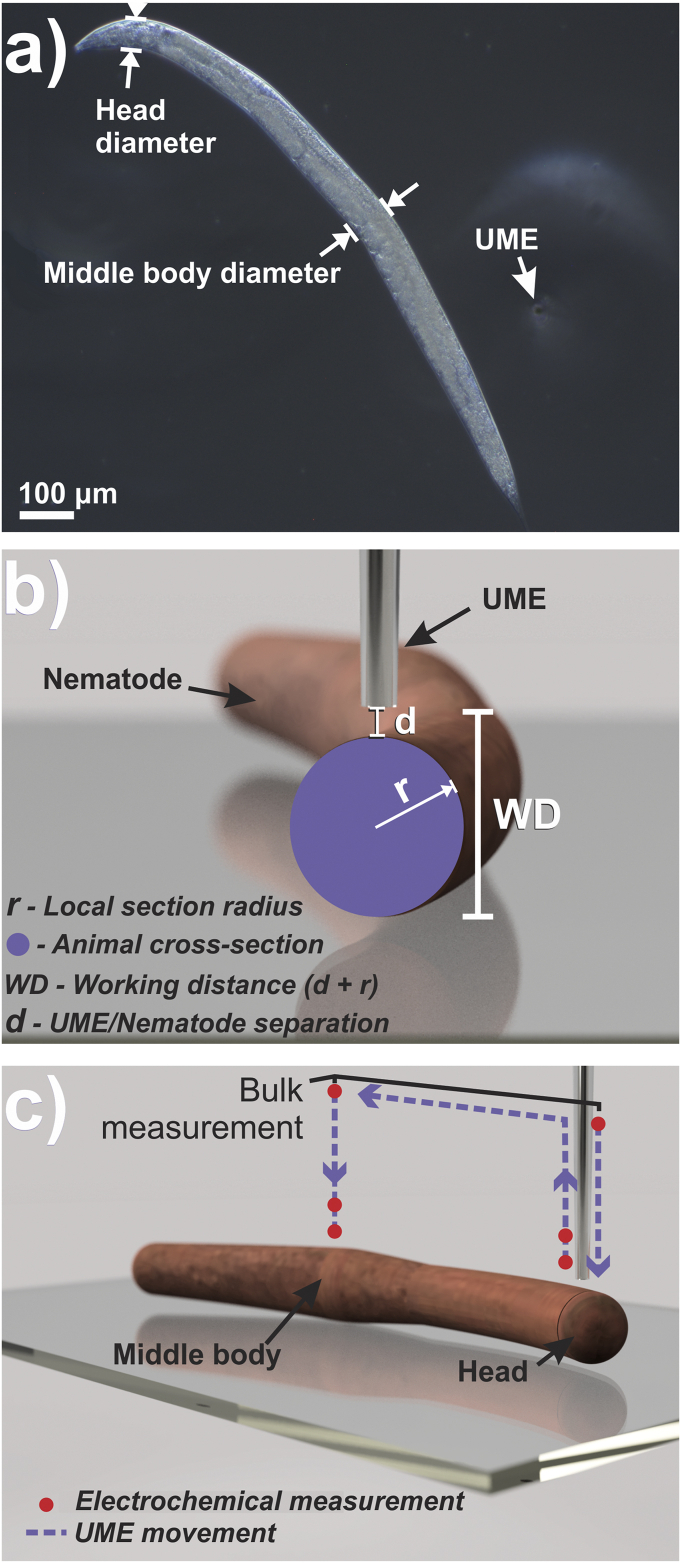
(a) Optical micrograph of a C. elegans worm showing the two regions of interest (head and middle body). The vulva was used as an anatomical reference for the middle body measurements. (b) Schematic representation of UME positioning for single-point measurements. The purple circle represents the cross-section of the worm body in the regions of interest. Measurements were performed at specific work distances (WD) calculated using the desired fixed distance (d) of 20 or 40 μm, considering the local worm thickness (twice its radius, r). (c) Representation of the UME movement on the single-point self-referenced measurement protocol. Dashed lines represent the UME path and red dots, the local electrochemical measurements. (For interpretation of the references to colour in this figure legend, the reader is referred to the Web version of this article.)

Local OCRs at different regions of the body of a worm were determined by single-point, self-referencing SECM measurements with the UME held at a selected *x-y* position and at a fixed distance from the nematode body. First, an approach curve (e.g. Figure S3) was recorded towards the Petri dish surface in the vicinity of the region of interest of the worm (the head and the middle body of a single *C. elegans,* Fig. 1a). Note that the worm’s vulva was used as an anatomic reference for the middle body, allowing single-point measurements to be performed at an equivalent location among different animals. The radius of the nematode’s cylindrical body was measured with the optical microscope (Fig. 1a) and used to set the UME working distance so that the electrode/nematode distance was first 40 μm, then 20 μm (Fig. 1b). As it will be discussed below (Section 3), at those distances, the UME measurements avoided starving the worm and the removal of oxygen from the animal by SECM-induced oxygen transfer [29], which would affect OCR calculations [30]. To a reasonable approximation, the UME is a probe of the local oxygen concentration with a spatial resolution of the size of the UME diffusion field (∼several μm) [38,39].

To reduce the effect of deterioration in the UME response during a series of measurements, which is a general issue for electrochemical measurements in biological media [[25], [40], [41], [42], [43]], a self-referencing method was employed, where the UME was retracted to the bulk solution (>500 μm) and the ORR current at bulk was recorded prior to the single-point measurements at the two heights (Fig. 1c). ORR limiting currents were used to determine the local OCR by FEM simulations using the software COMSOL Multiphysics 5.4 (for full model details see Section S-4 of the Supplementary Material). The calculated OCR values correspond to the animal’s average respiration rate in the time frame of the single-point measurements, approximately 6 s (see Section 2.3), and changes or fluctuations in respiration rates within this interval will not be detected by the measurements. Notwithstanding, under the experimental conditions employed herein (Section 2.2), the nematode’s respiration rate is expected to be constant in this time frame [8,9,44].

Experiments were performed and OCR values calculated for the head and vulva regions of single adult wild-type *C. elegans* grown in the absence (wild-type, see Section 2.2, herein referred to as only wt) and presence of 5-FU (wild-type grown with 5-FU, herein referred to as only wt-FU) and of sterile *glp-1* worms. For the wild-type worms, measurements were also performed in the presence of an ATP synthase inhibitor, *N*,*N*′-diciclohexilcarbodiimide (DCCD), which modulates mitochondrial respiration and thus induces a change in OCR. Measurements were recorded in M9 buffer solution in the presence of 20 μmol L^−1^ DCCD in 0.5% (v/v) dimetilsulfoxide (DMSO).

### 2.4 Quantification and statistical analysis

Local OCR values data are presented in graphs as mean ± standard error of the mean (S.E.M.). OCR values obtained at each fixed distance (40 and 20 μm) were used as duplicates in the statistical treatment. Sample sizes are reported in the figure legends. Statistical analysis was conducted using GraphPad Prism 8 software. Analyzes between the samples was performed via ANOVA with post hoc Tukey tests. P is significantly different for values lower than 0.05.

## 3 Results and discussion

### 3.1 Oxygen consumption is heterogenous along the *C. elegans* body

**Fig. 2:**
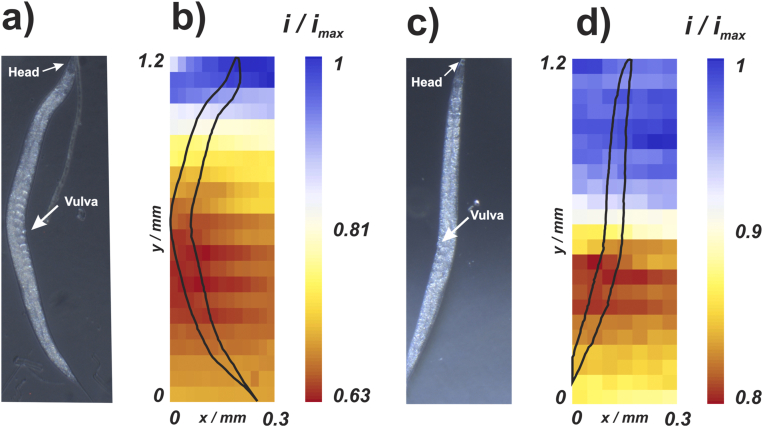
(a) Optical micrography and (b) SECM maps of a wild-type adult C. elegans. (c) Optical micrograph and (d) SECM map of a wild-type adult C. elegans grown in the presence of 15 μM of the DNA synthesis inhibitor 5-FU, resulting in dead embryos. Normalized oxygen reduction current maps correspond to diffusion-limited current, recorded at each x-y position with a platinized platinum UME. Reddish-orange and bluish-white regions indicate lower and higher ORR current values, respectively. The black lines in the SECM maps correspond to the worm outline deduced from the corresponding optical images. Distance between the UME and Petri dish surface: 100 μm. SECM image pixel size (x-y dimensions): (b) 25 by 50 μm and (d) 50 by 50 μm. Duration of the electrochemical measurement at each pixel: 6 s. Overall scan duration: (b) 140 min and (d) 145 min. (For interpretation of the references to colour in this figure legend, the reader is referred to the Web version of this article.)

SECM maps were recorded over anaesthetized adult wild-type worms, with an example shown in Fig. 2a. The normalized current values revealed a lower ORR current, indicative of lower local oxygen concentration, when the UME was in the vicinity of the middle region of the worm (reddish-orange regions in the maps) compared to the extremities, where higher ORR current values, indicative of higher local oxygen concentrations, were measured (bluish-white regions in the maps). The scan started at the top left and ended at the bottom right, and the ORR current in the worm’s middle region was approximately 60% of the maximum recorded current value at the start, suggesting that the middle body region uses more oxygen than other regions of the animal. The SECM maps clearly show a heterogeneous oxygen consumption pattern along the body of a *C. elegans*, which has not been reported before.

Anatomically, the middle body region concentrates much of the reproductive system, part of the intestine, and some muscle cells [45]. As reproduction was reported to be highly energy-consuming [31], we investigated the consequences of impairing part of the reproduction process on oxygen concentration along the worm’s body. For that, we killed embryonating eggs (adult animal in Fig. 2c) by growing the worms in the presence 5-FU, a DNA synthesis inhibitor that selectively affects rapidly dividing cells such as those from embryonating eggs [46]. The SECM map for a wild-type worm carrying dead eggs as a consequence of treatment with 5-FU revealed a pattern of oxygen concentration along the body similar to the one observed for wild-type worms carrying live embryos, with lower oxygen concentrations detected in the middle body compared to other body regions (Fig. 2d). However, the normalized current in the middle body region of 5-FU treated worms was approximately 0.8 (Fig. 2d), whereas the current measured in control worms was approximately 0.6 (Fig. 2b), indicating that the viability of eggs has a significant impact on middle body oxygen consumption. In both maps, the oxygen concentration gradient, resulting from the animal’s respiration, extended a considerable distance from the worm body, resulting in the large oxygen concentration boundary layer clearly seen in Fig. 2.

While the SECM maps in Fig. 2 suggest that OCR is heterogeneous along the body of a single worm and that embryonating eggs inside the uterus are major contributors to middle body oxygen consumption, there are some important technical aspects with these measurements that have to be considered:

(i) the middle body region has a larger diameter than the rest of the body, and, consequently, the UME/worm distance is smaller in this location compared to other regions, as all scans were performed at a fixed working distance with respect to the Petri dish (see Section 2.3.1);
(ii) there is some deterioration in the UME response for ORR during the scan, as can be discerned from the normalized current, which (away from the worm) is close to 1 towards the start of the scan, but < 1 towards the end (Fig. 2b and d). An estimation of the deterioration of the UME response during imaging can be made by taking the ratio of currents in the beginning and final lines at pixels away from the nematode, since in both cases the nematode region diameter is smaller and so the current approaches to the bulk value. For the data in Fig. 2b, the current deteriorates by approximately 30% and for the data in Fig. 2d the deterioration is 15%;
(iii) The long SECM imaging times, approximately 140 min even at this low spatial resolution, could lead to animal stress, affecting its metabolic activity. It is important to note that despite the different pixel sizes, both images have similar scan times (∼140 min), achieved by increasing the waiting time between image pixels in Fig. 2d (compared to 2b).

To overcome these problems and put the measurements on a quantitative footing, we adopted a single-point approach where measurements were restricted to two regions of interest: the head and the region near the vulva, with self-referencing (calibration) of the response for each and every measurement (*vide infra*). This enabled measurements on a large population and provided robust statistical analysis. To conduct these experiments, we developed a quantitative model for OCR measurements.

### 3.2 Quantitative local OCR measurements

#### 3.2.1 FEM model for local OCR

As seen in Fig. 2, the OCR over the nematode body cannot be approximated to be homogenous. To obtain quantitative local OCR data from the SECM single-point measurements, FEM simulations were used. To account for the complexity of the heterogenous OCR distribution along the nematode body and its irregular shape, a 3D model without symmetry planes would be needed, but these are computationally very expensive, particularly for high-throughput analyses [47], as employed here (note that every worm has a slightly different geometry). Although the entire nematode body affects the oxygen boundary layer around the animal, it is reasonable to assume that the local OCR of a specific region will have a larger bearing on the local oxygen concentration around this particular region. Therefore, we adopted a 2D axis-symmetric model, consisting of a sphere with the same diameter as the animal’s cross-section in the regions of interest (head and vulva regions). With the UME positioned above at the two electrode/surface separations used in the experiments, the impact of the animal’s respiration on the UME current response was investigated.

The difference between the two geometries was analyzed by simulating the current response at the UME positioned 20 and 40 μm from the center point of a spherical (2D axis-symmetric model) and a cylindrical (3D model) substrate consuming oxygen at the same rate using a simplified 3D model (Section S-5 of the Supplementary Material). OCR is dependent on the investigated surface area and the UME response is mostly dominated by the O_2_ consumption of regions of the substrate near the electrode (Figure S6). As simulating a sphere of radius ***r*** in a 2D axis-symmetric space approximates to a 3D model of a cylinder of radius ***r*** and twice the length (Figure S6), the 2D axis-symmetric model is a reasonable approximation for quantitative local OCR measurements.

#### 3.2.2 Calibrating UME currents to local OCR values

To obtain quantitative data on the local OCR, FEM analysis was performed on a geometry that reflected the experiments, as discussed above. Respiration was simulated as a flux of oxygen from the solution towards the worm’s surface. This flux ($J_{O_{2}}$) is driven by a rate constant, ***k***_***Resp***_, and is described by Equation (1):

(1) $$J_{O_{2}}=\;k_{Resp}\cdot C_{O_{2}}$$

where $J_{O_{2}}$ is the molecular flux of oxygen, ***k***_***Resp***_ is the rate constant, and $C_{O_{2}}$is the local oxygen concentration at the worm/solution interface. Simulations were performed for a range of ***k***_***Resp***_, representing possible respiratory rates. Section S-4 of the Supplementary Material has further details on this FEM model.

**Fig. 3:**
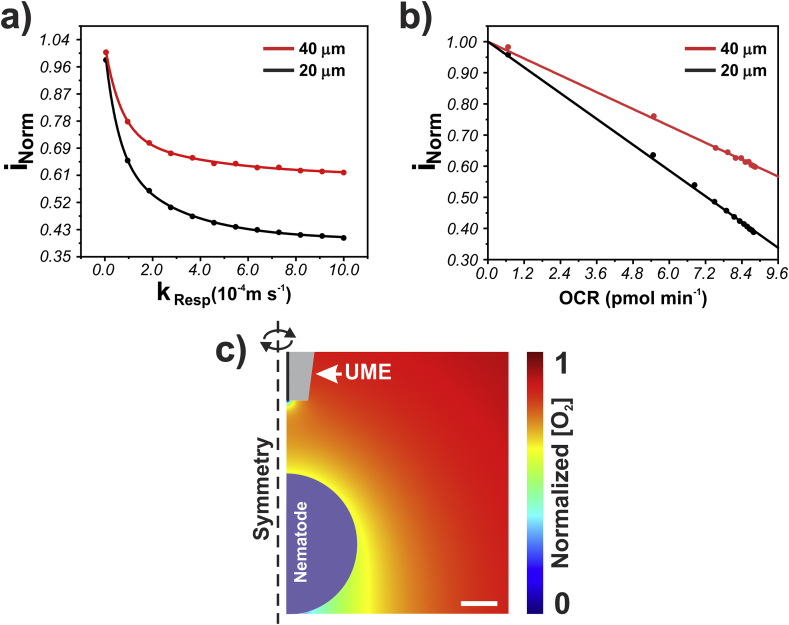
(a) Typical working curve showing the normalized UME current dependency on the rate constant **k**_**Resp**_, representing different respiratory rates. (b) Typical calibrations plots of local normalized UME current vs OCR for the two UME/worm surface separations. (c) Oxygen concentration profile around the worm (sphere) and over the UME positioned 20 μm over the head region with an assumed OCR of 6 pmol min^−1^. Scale bar = 10 μm.

The normalized (self-referenced) experimental currents obtained using the platinized platinum UME were matched to the simulated normalized currents in ***i***_***Norm***_ vs ***k***_***Resp***_ plots (Fig. 3a), to find the local respiratory rate. OCR can be calculated from the local respiratory rate by integrating the resulting oxygen flux, driven by ***k***_***Resp***_, over the investigated region of the worm. The resulting OCR values were plotted against normalized UME current, generating calibration plots of ***i***_***Norm***_ vs OCR (Fig. 3b).

To highlight the relatively non-invasive nature of these measurements, Fig. 3c shows an O_2_ concentration map at the closest UME/substrate separation of 20 μm for an equivalent OCR of 6 pmol min^−1^ at the spherical substrate. Importantly, even at this close distance, the UME electrochemical process does not perturb the O_2_ flux to the spherical object (worm) as the UME diffusion layer does not reach the worm surface (Fig. 3c). In fact, the UME can be considered a sensor of the local O_2_ concentration boundary layer around the worm, justifying our choice of relatively large working distances. The adopted distances eliminated any SECM-induced oxygen transfer caused by the UME [29,30], while keeping the electrode in a region where it is sensitive to changes at the O_2_ boundary layer around the worm. A further consequence here is that the UME electroactive disk’s geometry does not need to be precisely known, especially as measurements are made in a self-referencing format. In this case, the UME current ratio at 20 and 40 μm away from the animal surface can be considered to be the local concentration ratio at these positions. Indeed, if the UME is truly passive, and does not disturb the boundary layer composition by consuming oxygen or shielding the substrate from bulk solution O_2_, simulations can be made without it, as discussed in Section S-6 of the Supplementary Material. Analysis of the experimental data using this simplified approach yielded OCR values with a difference of approximately 15% compared to the more detailed models (where the UME geometry is considered), and can be used for quick OCR estimations or in simple systems (see Section S-6).

### 3.3 Local OCR measurements reveal high reproductive system oxygen consumption rates

**Fig. 4:**
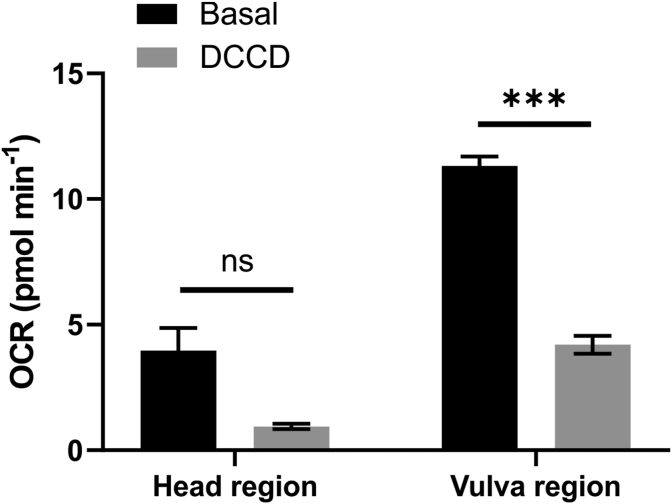
Experiments were performed in the absence (basal, n = 6) or presence of 20 μM ATP synthase inhibitor DCCD in 0.5% DMSO (DCCD, n = 2). Results are presented as mean ± S.E.M.. Two-way ANOVA with post hoc Tukey test is significantly different for p < 0.05. For head vehicle vs. head DCCD, p = 0.1866; for vulva vehicle vs. vulva DCCD, p = 0.0006; for head DCCD vs. vulva DCCD, p = 0.2957; for head vehicle vs. vulva vehicle, p < 0.0001.

Local ORR current values were obtained using the single-point self-referencing method as described in Section 2.3.2 and were used *in tandem* with the FEM simulation to calculate the local OCR, as outlined above in section 3.2 and Section S-4. The OCR values obtained for the head and vulva regions of wild-type adult *C. elegans* were 4.0 ± 2.3 pmol min^−1^ and 11.3 ± 1.0 pmol min^−1^, respectively (Fig. 4), confirming the heterogeneous oxygen consumption along the worm body, and explaining the high contrast between the two regions detected in the SECM images (Fig. 2). The local middle body OCR value is similar to reported whole-worm OCR (15 pmol min ^−1^), measured by bulk [8], and microfluidic methods [9], for animals in the same age and conditions. This showcases how bulk measurements are blind to single animal heterogeneities and that current OCR measuring methods are mostly reporting the middle body region respiration. The latter is important as, being a proxy for metabolic activity, OCR measurements are routinely used to assess toxicity of new substances [[48], [49], [50]]. If the tested substances have little impact on the middle body respiration but impact other regions, this would be hardly detected by bulk measurements.

We demonstrate the effects of pharmacological treatments on local OCRs by modulating mitochondrial activity. OCRs in living eukaryotic organisms are predominantly determined by mitochondrial respiration. Thus, interfering with mitochondrial activity will translate into changes in OCRs. Numerous chemical compounds are well-established modulators of mitochondrial activity, including DCCD, an ATP synthase complex inhibitor [44,51]. Under physiological situations, ATP synthesis and oxygen consumption resulting from the transport of electrons through the mitochondrial electron transport chain are coupled processes, in a manner that inhibition of one will inhibit the other [52]. We used DCCD to inhibit ATP synthase in live worms and assessed the consequences on local OCR using the single-point measurement strategy. In the presence of the inhibitor DCCD, OCR values in the head region decreased by 76%, while in the vulva region (middle body), they decreased by 63% (Fig. 4), suggesting the measured OCR is a consequence of local mitochondrial activity. Bulk measurements performed with DCCD over a population of worms found a whole-worm OCR decrease (65%) [44] similar to what we observed at the middle body region (63%).

**Fig. 5:**
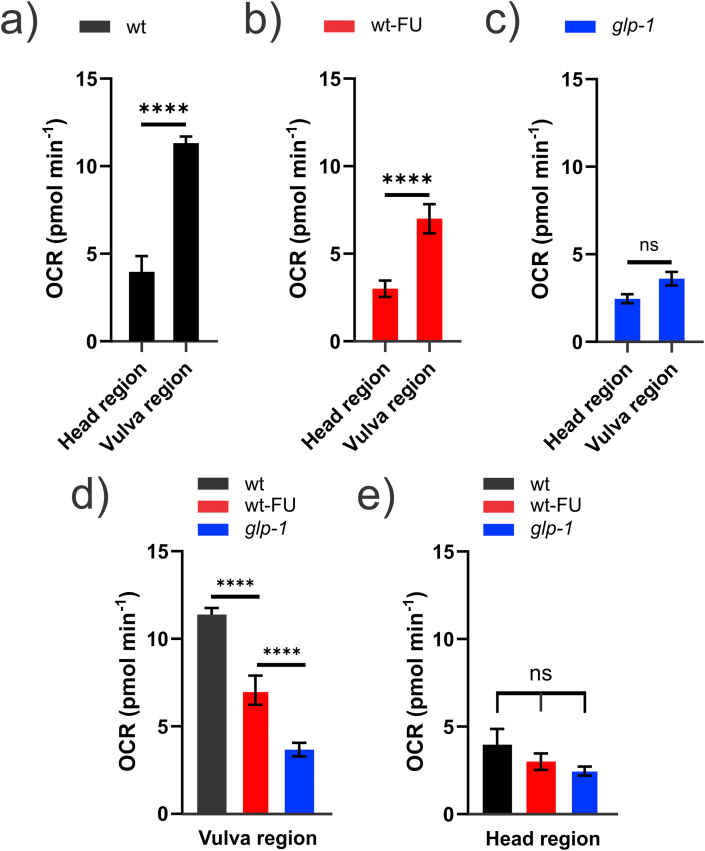
Wild-type animals grown in the (a) absence (wt, n = 6) or (b) presence of 5-FU (wt-FU, head region n = 11, vulva region n = 12) and (c) germline-less glp-1 worms (head region n = 16, vulva region n = 13). (d) and (c) show the comparison between the vulva and head region of the 3 different groups, respectively. For all panels: wt = fertile worms, wt-FU = worm carrying dead embryos, and glp-1 = worm with an empty gonad. Bars represent mean ± S.E.M. Two-way ANOVA with post hoc Tukey test is significantly different for p < 0.05. ∗∗∗∗ is p < 0.0001.

Results obtained by SECM mapping in the presence of 5-FU (Fig. 2d) suggest that developing eggs consume a significant amount of oxygen, a finding confirmed by OCR values obtained by SECM single-point measurements (Fig. 5). The OCR value calculated for the vulva region of worms grown in the presence of 5-FU was 7.0 ± 1.8 pmol min^−1^, which represents a decrease of 38% in oxygen consumption, when compared to the same region of control worms (Fig. 5a and b), reinforcing the idea that developing eggs are important contributors in the metabolic activity of an adult hermaphrodite worm. Whole-worm OCR obtained by bulk methods for the same condition is reported to be approximately 9 pmol min ^−1^ [8]. When compared to the local OCR values we obtained for the head and vulva region (Fig. 5b), it is clear that bulk OCR measurements are dominated by the middle body region respiration and are not sensitive to the respiration of other regions, such as the head.

The nematode reproductive system extends far beyond embryonating eggs to include a proliferating germline and the somatic gonad, which may also have high metabolic activity. To assess the impact of the gonad on local OCRs, we performed measurements on the *glp-1* strain, which contains a temperature-sensitive allele that when activated by growth of the worm at a restrictive temperature, impairs the proliferation of the germ line, resulting in a sterile worm with an empty gonad [53]. OCR values obtained for *glp-1* were 2.5 ± 0.6 pmol min^−1^ for the head region and 3.6 ± 0.8 pmol min^−1^ for the vulva region (Fig. 5c). The absence of germline, oocytes, sperm, and eggs in *glp-1* significantly reduced OCR values for the vulva region, abolishing the difference between head and vulva OCRs detected in the other experimental groups (compare Fig. 5c with b and a). OCR values for the vulva region in *glp-1* were also 30% lower than those for the same region in wild-type worms carrying dead embryos, indicating that the germline, oocytes, and sperm cells also contribute to the global OCR of the vulva region (Fig. 5d). Interestingly, OCR values measured in the head region were similar for all the experimental groups (Fig. 5e), confirming that the OCR differences in the vulva region are associated with differences in the reproductive system and the treatments have little or no effect in the middle body somatic cells metabolism. Although 5-FU and *glp-1* effects are highly selective to the reproductive system, the possibility of measuring local OCRs at different tissues, *in vivo*, opens an interesting avenue for using SECM single-point measurements to effectively quantify the toxicity and impact of different pharmacological treatments to the metabolic activity of different anatomical sections of an organism.

**Fig. 6:**
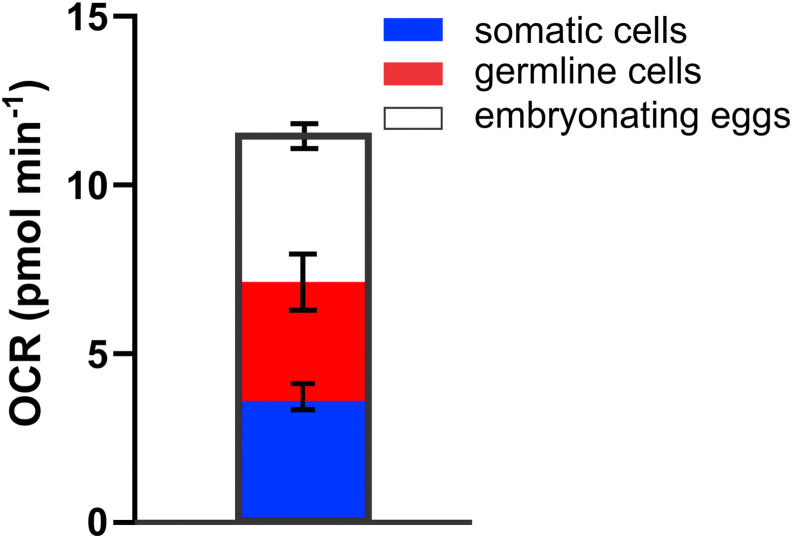
Represented OCR values were obtained for adult hermaphrodites under control conditions (wild-type, n = 6), conditions leading to dead embryos (wt-FU, n = 12), and conditions leading to an empty gonad (glp-1(e2144ts) worms, n = 13). Bars represent mean ± S.E.M..

Further, we can use the local OCR values obtained after pharmacological (5-FU) or genetic (*glp-1*) interventions to estimate the relative contributions of the reproductive system components toward the global oxygen consumption in the vulva region of hermaphrodite worms. The data suggest that 68% of the oxygen consumed in the vulva region is associated with reproductive activity, with embryonating eggs responsible for 38% and germline cells for 30% of oxygen consumption (Fig. 6). These findings reinforce the idea that reproduction in adult *C. elegans* is an energetically-demanding process [31].

## 4 Conclusions

By employing SECM as a tool for probing local oxygen concentrations, we were able to visualize and quantify heterogeneous respiration rates along the body of live adult *C. elegans.* SECM self-referencing single-point measurements are particularly powerful for space-resolved measurements, as the SECM tip electrode response is essentially calibrated before every measurement. Moreover, by focusing on two regions of interest on the worm body, it was possible to make measurements on a large sample, thereby allowing a robust statistical analysis of the data. The middle body region was found to have a OCR 3 fold that of the head region and we were able to show clearly that reproductive activity is a major contributor (68%) for middle body oxygen consumption in *C. elegans,* highlighting the high energy demand of reproduction*.* This is the first time that heterogenous OCR along a *C. elegans* is visualized, quantified and reported. By comparing local OCRs with whole-worm values obtained by bulk measurements we demonstrate that commonly employed methods are blind to the respiration of other anatomic regions in the animal than the middle body. These findings demonstrate that SECM, and specifically the single-point self-referencing measurements *in tandem* with FEM simulation approach, is able to detect subtle region/tissue-specific differences in respiration in live worms. Although the present work used *C. elegans* as a model, this approach is well-suited to probe oxygen consumption rates in virtually all anatomical parts/tissues of small organisms. This work thus presents a road map for the study and analysis of respiration in small organisms using SECM, which can be extended to metabolism, toxicity and pharmacological developments.

## Notes

The author declares no competing financial interest.

## CRediT authorship contribution statement

**Carla S. Santos:** Conceptualization, Data curation, Visualization, Formal analysis, Investigation, Methodology, Writing - original draft, Writing - review & editing. **Felipe Macedo:** Investigation, Writing - original draft, Writing - review & editing. **Alicia J. Kowaltowski:** Formal analysis, Supervision, Resources, Writing - original draft, Writing - review & editing. **Mauro Bertotti:** Conceptualization, Formal analysis, Supervision, Writing - original draft, Writing - review & editing. **Patrick R. Unwin:** Visualization, Formal analysis, Supervision, Resources, Writing - original draft, Writing - review & editing. **Fernanda Marques da Cunha:** Formal analysis, Supervision, Resources, Writing - original draft, Writing - review & editing. **Gabriel N Meloni:** Conceptualization, Data curation, Visualization, Formal analysis, Software, Methodology, Writing - original draft, Writing - review & editing.

## Declaration of competing interest

The authors declare that they have no known competing financial interests or personal relationships that could have appeared to influence the work reported in this paper.
