# Supplementary material for: Unveiling the contribution of the reproductive system of individual Caenorhabditis elegans on oxygen consumption by single-point scanning electrochemical microscopy measurements
Source: Anal Chim Acta. 2021 Feb 15;1146:88–97. doi: 10.1016/j.aca.2020.12.030 (PMC7836392; doi:10.1016/j.aca.2020.12.030)
Supplement: Multimedia component 1 [file mmc1.pdf]

Supplementary Material

Unveiling the contribution of the reproductive system of individual *Caenorhabditis elegans* on oxygen consumption by single-point scanning electrochemical microscopy measurements

Carla Santana Santos,[a, 1,] * Felipe Macedo,[c] Alicia J. Kowaltowski,[b] Mauro Bertotti,[a] Patrick R. Unwin,[d,e] Fernanda Marques da Cunha,[c] Gabriel N. Meloni [d,e]*

[a] Departamento de Química Fundamental, [b] Departamento de Bioquímica, Instituto de Química, Universidade de São Paulo, Av. Professor Lineu Prestes, 748, 05508-000, São Paulo, SP, Brasil.

[c] Departamento de Bioquímica, Escola Paulista de Medicina, Universidade Federal de São Paulo, Rua três de maio, 100, 04044-020, São Paulo, Brasil.

[d] Department of Chemistry, [e] Bio-Electrical Engineering Innovation Hub, University of Warwick, Gibbet Hill Road, CV4 7AL, Coventry, United Kingdom.

*Correspondence: carla.santanasantos@rub.de, Gabriel.Meloni@warwick.ac.uk

[1]**Current address:** *Analytical Chemistry−Center for Electrochemical Sciences (CES), Faculty of Chemistry and Biochemistry, Ruhr-Universität Bochum, Universitätßtrasse 150, D-44780 Bochum, Germany*

Content

S-1 Worm images……………………………………………………………………S-3
S-2 SECM tip fabrication and characterization……………………………….........S-4
S-3 SECM approach curve…………………………………………………………...S-6
S-4 Finite Element Method (FEM) model and simulation parameters……………………S-7
S-5 Comparison between 2D axis-symmetric and 3D models…………………………….S-10
S-6 UME as a local oxygen concentration probe …………………………………………S-12

S-1 Worm images

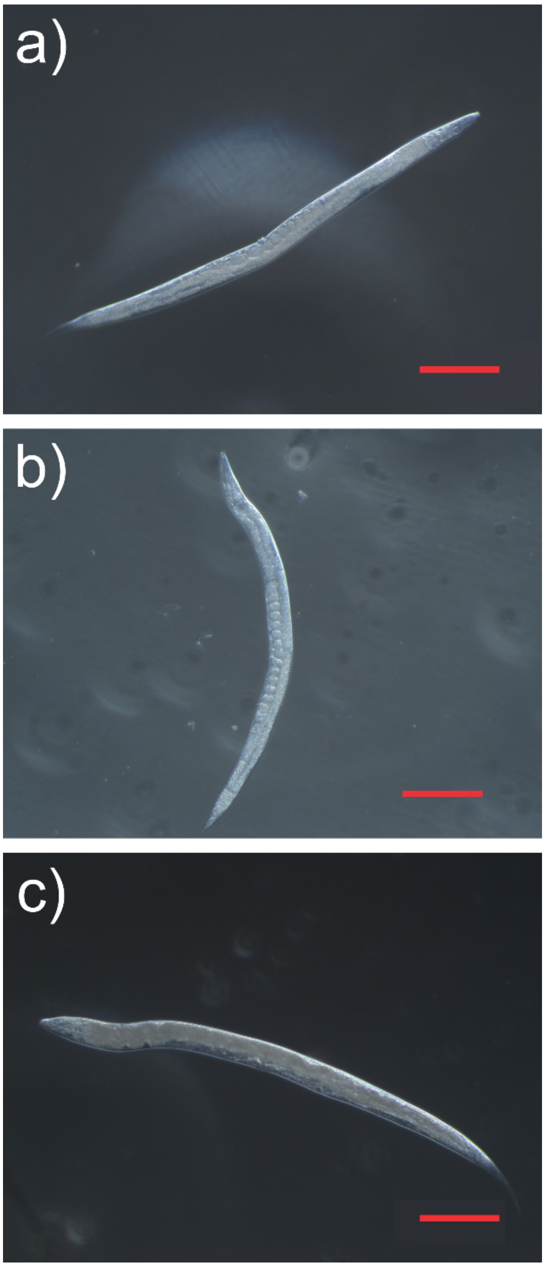

Figure S1. *Optical images acquired by an inverted microscope in the SECM setup showing a) wild-type adult C. elegans, b) wild type adult C. elegans grown in the presence of 15 µM of the DNA synthesis inhibitor 5-FU, c) germline-less (sterile) adult glp-1(e2144ts) worms. Scale bar: 200 µm.*

S-2 SECM tip fabrication and characterization

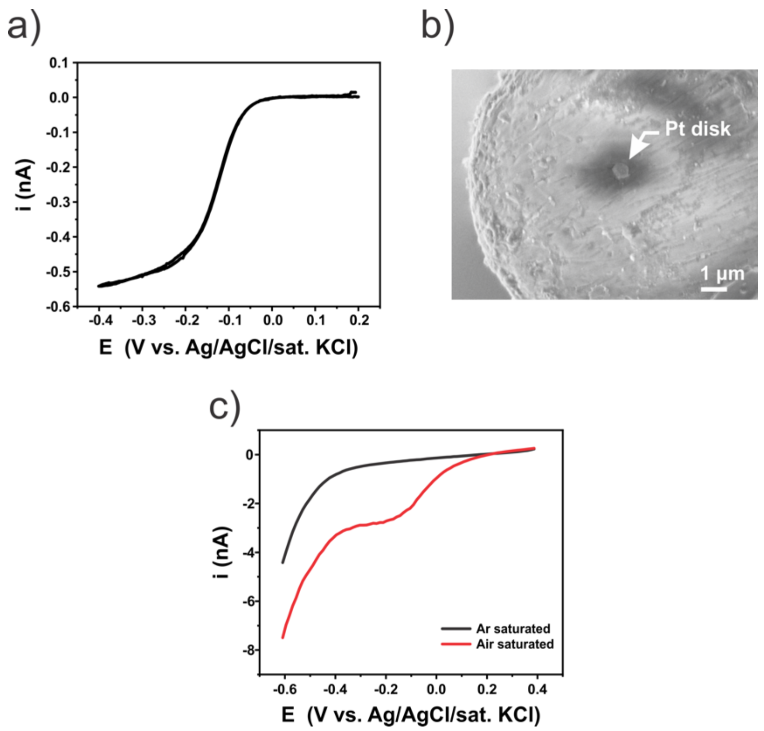

Figure S2. *a) Representative cyclic voltammogram recorded at a platinum UME in 5 mmol* L^{-1} *[Ru(NH)$_6$Cl$_3$] in 100 mmol* L^{-1} *KCl solution, before platinization (radius = 500 nm, RG = 20). b) Scanning electron microscopy of a freshly fabricated and unpolished UME. Image used to evaluate the fabrication parameters. Electron accelerating voltage = 3 kV. c) Representative line scan voltammograms recorded with a platinized platinum UME in an argon (Black) and air (Red) saturated M9 solution (effective radius = 2.5 µm). Scan rate = 0.1 V* s^{-1}.

Platinum ultramicroelectrodes (UMEs) used as the scanning tip of a scanning electrochemical microscope (SECM) were prepared from 50 µm diameter platinum wire (hard, 99.99% purity, Goodfellow) and quartz capillary tubes (ID: 0.3 mm, OD: 1 mm, Sutter Instruments) using a laser capillary puller (P-2000, Sutter Instruments). Fabrication followed reported standard protocols.[1] Prior to the platinization process, UMEs were characterized by recording cyclic voltammograms for the one-electron reduction of 5 mmol L^{-1} hexaamineruthenium (III) chloride ([Ru(NH$_3$)$_6$Cl$_3$], CAS 14282-91-8, Alfa Aesar) in 100 mmol L^{-1} potassium chloride solution (KCl, CAS 7447-40-7, Sigma); Figure S2a. Diffusion-

limited [$Ru(NH_3)_6^{3+}$] reduction current was used to calculate the UME radius, assuming a planar disk geometry.[2,3] All UMEs presented a radius between 500 and 700 nm (Figure S2b). The radius of the glass sheet surrounding the disk electrode, known as ***Rg*** (radius of glass),[4] was measured by optical microscopy and found to be approx. 10 μm for all UMEs. The platinization processes was designed to increase the electrochemical response of the UME (increased signal-to-noise ratio) without drastically changing its geometry. It followed a procedure previously reported by us,[5] allowing for the active area to be approximated to a planar disk. This is important to facilitate the representation of the UME's geometry in finite element method (FEM) models without the need for electron microscopy techniques to characterize the true geometry of the UME surface.[6,7] As all studies were performed with normalized current values (self-referencing, see main manuscript), and the UME size is small (see below), the choice of the approximated UME geometry does not impact the final result. Briefly, the platinization processes consisted in cycling the UME potential between 0.3 V and – 0.5 V (*vs* Ag/AgCl/sat. KCl at a sweep rate of 0.100 V s^{-1}) in a 1 mmol L^{-1} hexachloroplatinic acid hexahydrate ($H_2PtCl_6 \cdot 6H_2O$, CAS 18497-13-7, Sigma) in 0.5 mol L^{-1} H_2SO_4 solution. The potential sweep was repeated 30 times. A representative voltammogram for oxygen reduction at a platinized UME is shown in Figure S2c and suggests a 5-fold increase in effective radius (assuming a planar disk geometry). After platinization, UMEs presented a 3 to 5-fold increase in effective radius with resulting radii values between 1 and 2.5 μm. As discussed in the main text, the actual electrode geometry for these measurements does not have a large bearing on the analysis as long as the diffusion field at the UME is small compared to that at the nematode, so that the UME approximates to a point probe of concentration.

S-3 SECM approach curve

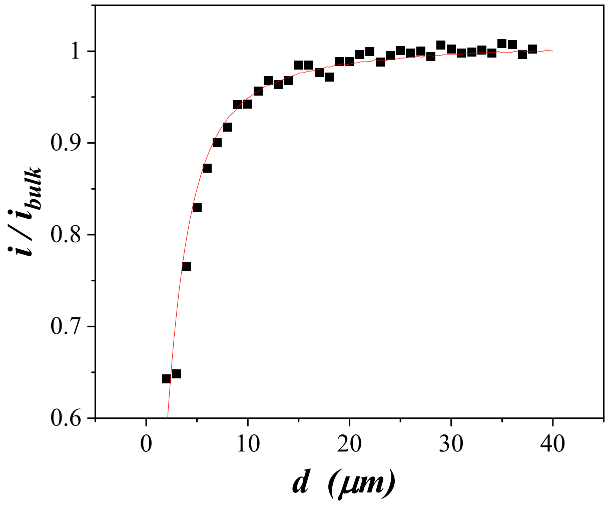

Figure S3. *Approach curve for ORR in aerated solution recorded using a platinized platinum UME (effective radius = 2 µm, Rg = 10 µm) towards a Petri dish surface in M9 solution. Scatted plot: experimental data. Red line: simulated approach curve towards an insulating substrate using the real UME geometry, captured during the characterization procedures. Scan increment: 1 µm. Approach curve speed: 10 µm/s. UME potential = – 0.4 V vs Ag/AgCl/sat. KCl. UME minimum approach distance: 2.5 µm.*

Figure S3 shows a representative hindered diffusion approach curve of the type obtained prior to SECM experiments presented in the manuscript, and the typical UME current dependence on the electrode/substrate separation can be observed. The approach curve was recorded using a platinized platinum UME at oxygen reduction reaction (ORR) conditions (–0.4 V *vs* Ag/AgCl/sat. KCl), where current is limited by oxygen diffusion. Currents were normalized by the value obtained in bulk solution. The UME is moved towards the insulating Petri dish surface at a constant speed while the current is recorded. At a given Z- distance (dependent on electrode size and geometry),[4] the normalized current starts to decrease due to hindrance of oxygen diffusion towards the UME caused by the small electrode/substrate gap.[8] By analyzing results shown in Figure S3, we conclude that the recorded current approximates to that recorded in bulk solution at the adopted largest working distance for the SECM map experiments (100 µm – Figure 2 in the main manuscript) .

S-4. Finite Element Method (FEM) model and simulation parameters

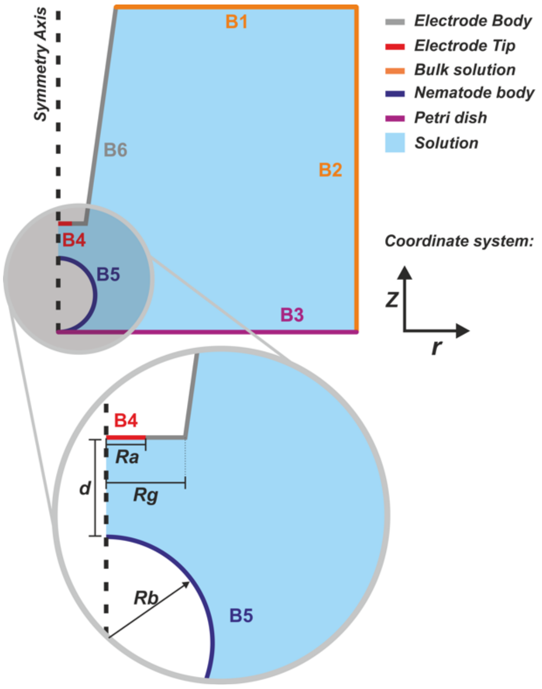

Figure S4. *Schematic of the simulation domain used for the FEM model. Boundary conditions are specified in table S1. Drawing not to scale.*

Table S1. *Summarized boundary conditions for the FEM model. Only fluxes normal to the boundary were considered, noted by "**n**".*

**Boundary**	**Flux / Concentration condition**
B1	$\boldsymbol{n \cdot J_i = 0}$
B2	$[O_2] = 277\ \mu M$ [9]
B3	$\boldsymbol{n \cdot J_i} = 0$
B4	$[O_2] = 0$
B5	$\boldsymbol{n} \cdot J_{O_2} = -k_{Resp}[O_2]$
B6	$\boldsymbol{n} \cdot J_i = 0$

Table S2. *Experimentally fitted parameters for the FEM model*

**Parameter**	**Description**
R_a	UME effective radius
d	Separation between the UME and nematode body
Rg	Radius of the UME counting the outer glass sheet
R_b	Radius of the cross-section of the nematode body

Table S3. *List of parameters used in all simulations (not including experimentally-fitted data described in Table S2).*

Symbol	Value	Description
n	*4*	Number of electrons involved in the charge transfer reaction
d	*20 and 40* μm	UME /Nematode separation
C_{O_2}	*277* μM	Oxygen concentration at bulk solution
D_{O_2}	$2.2 \times 10^{-5}\ cm^2\ s^{-1}$ [10]	Oxygen diffusion coefficient

Simulations for the UME response near different sections of the nematode body were performed according to the experimental framework employed and described in the manuscript. The simulation domain employed is portrayed in Figure S4, where the UME body (B6), electroactive surface (B4) and the nematode body cross section (B5) can be seen. Simulation domain size (> 500*$\boldsymbol{R_b}$ in both r and z dimensions) and mesh density (> 100.000 elements) were set as such that simulation results were independent of both. Boundary conditions for every boundary in the domain are specified in table S1. $\boldsymbol{R_b}$ was extracted from optical images from the animal's body (Figure 1 of the manuscript and Figure S1), which were recorded with an inverted optical microscope attached to the SECM equipment. The value of ***d*** was set to 20 and 40 μm. $\boldsymbol{R_a}$ was calculated using the ORR limiting currents recorded in bulk solution and assuming a planar disk geometry. ***Rg*** was calculated from optical images.

Simulations were performed in a 2D axis-symmetric geometry in COMSOL Multiphysics 5.4 using the transport of diluted species module using the stationary solver. Mass transport is described by diffusion only, assuming no migration due to the high electrolyte concentration in solution (M9 buffer solution). The flux ($\boldsymbol{J_i}$) of each chemical species ***i*** is described by equation S1.

$$\boldsymbol{J_i} = -D_i \nabla C_i \qquad \text{(S1)}$$

For simulating the current at the UME, a simple 4-electron O_2 reduction process at the platinum interface was assumed, as described in equation S2.[11,12]

$$O_2 + 4H^+ + 4e^- \leftrightharpoons 2H_2O \qquad \text{(S2)}$$

At the UME surface (boundary B4) a concentration boundary condition of $[O_2] = 0$ was in place to simulate the diffusion-limited ORR. The current at the UME was calculated by integrating the molecular flux of oxygen over the boundary B4.

Respiration by the animal was simulated by applying an inward oxygen flux at the animal surface (B5), as described in Table S1. Simulations were performed for varying values of $\boldsymbol{k_{Resp}}$ at the boundary, representing different possible respiration rates, and the impact of the O_2 depletion layer created by boundary B5 on the UME response (B4) was investigated. Experimental diffusion-limited currents for oxygen reduction at the UME near the worm were normalized ($\boldsymbol{i_{Norm}}$) by current values recorded in bulk and fitted to $\boldsymbol{i_{Norm}}$ *vs.* $\boldsymbol{k_{Resp}}$ plots, as seen in Figure S5a.

Oxygen consumption rate (OCR) values were then calculated by integrating the O_2 flux at B5. Figure S5b shows the relation between $\mathbf{k_{Resp}}$ and OCR for the head region of one animal simulated as a 20 μm radius sphere (see Section SI-5 below). Calibration curves of $\mathbf{i_{Norm}}$ vs OCR were constructed (Figure 3b, main manuscript) and used to find the local OCR. This was performed for every individual single-point measurement as it depends on region thickness.

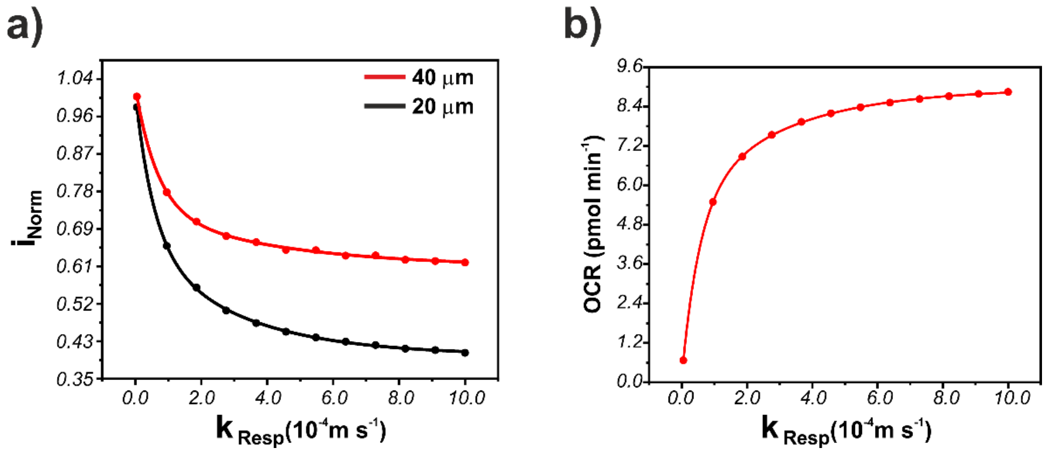

Figure S5. *Typical set of graphs generated by FEM simulation for each single-point SECM measurement, showing the effect of the rate constant applied to boundary B5 for the inward oxygen flow at (a) the UME current for the two UME/nematode separations (black, 20 and red 40 μm) used and (b) the resulting oxygen consumption rate (OCR).*

The $\boldsymbol{k_{Resp}}$ values used in the simulations were chosen empirically by simulating a large range (50 distinct values spanning from $1 \times 10^{-8}\ m\ s^{-1}$ to $10\ m\ s^{-1}$) for the first sets of data (animals) examined. Further simulations where performed using 13 distinct $\boldsymbol{k_{Resp}}$ values (from $1 \times 10^{-8}\ m\ s^{-1}$ to $1 \times 10^{-3}\ m\ s^{-1}$), covering all the respiratory rates encountered.

S-5 Comparison between 2D axis-symmetric and 3D models

The simulation geometry used (2D axis-symmetric) for the FEM model does not allow for the real animal geometry to be represented (irregular cylinder with varying radius). A full 3D model, without symmetry planes, is necessary to represent the true geometry of the system, which would greatly increase the computational cost and time. The computational price penalty for 3D modelling is particularly prohibitive for high-throughput applications such as the one presented in the manuscript. In the model used for all simulations in the manuscript, the substrate (representing the animal) was assumed to be a sphere with the same radius as the radius of the region of interest of the animal. This is a simplification allowing for the simulations to be performed in 2D. To evaluate the impact of this compromise on the simulation results, a simplified 3D model of a cylinder, representing the worm, was compared to a 2D axis-symmetric model of a spherical substrate. For both substrates, the concentration of O_2 at the surfaces was set to 0 (diffusion limited oxygen consumption) and the O_2 concentration at a separation of 20 and 40 μm from the center point of both substrates (Figure S6a), representing the position of an UME, was calculated and normalized to bulk concentration values. The models were run for different cylinder lengths (*h*) and a fixed cylinder and sphere radius (*r*). Normalized current ratios for a UME positioned at the concentration probe locations were calculated from the normalized O_2 concentrations (see Section SI-6 below) and the currents for the cylinder model were divided by those from the

spherical one (Figure S6b) to demonstrate how the values for the normalized UME current compare between the 2 models.

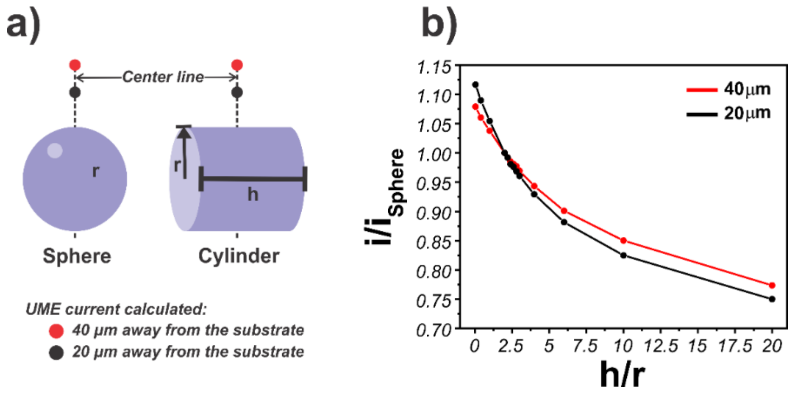

Figure S6. *Comparison between a 2D axis-symmetric sphere and 3D cylinder geometries FEM models. a) Representation of the two geometries showing the geometric descriptors "r" and "h", the center line of the geometry and the locations where O_2 concentrations were probed at 40 (red) and 20 µm (black). b) Comparison between the current ratios at a UME positioned at the concentration probe locations, calculated from the concentration ratios between the probe points (20 and 40 µm) and bulk values, for a sphere and a cylinder with different h/r ratios.*

Although longer cylinder lengths impact more the current sampled at the center of the substrate (for both working distances), it is clearly that the UME response is dominated by the O_2 consumption of regions of the substrate near the electrode, equivalent to smaller *h/r* values in Figure S6. As the cylinder gets longer, its length has a diminishing effect on the normalized current ratio, discerned by the lower slope of the curve at larger *h/r* ratios (longer cylinders – ca. 2.5). Even assuming a homogeneous O_2 consumption along the substrate, the length (*h*) has a smaller bearing on the O_2 concentration boundary layer than the radius (*r*). The intersection point between the two lines in Figure S6b ($i/i_{Sphere} = 1$) represents the case for a cylinder of *h/r* = 2, which equates in surface area to a sphere of radius *r*. At this point the 3D and 2D axis-symmetric model are equivalent, despite the distinct geometry.

If the nematode geometry would be simplified to a constant radius cylinder it would have a *h/r* ratio between 15-20 (depending on the animal shape, the "average" radius can vary). Hence approximating the substrate to a sphere would represent at most a difference of <25% between

the 2 geometries (Figure S6b). This is the most these 2 models differ, as it assumes homogenous O_2 consumption along the substrate, which is not the case for *C. elegans* (Figure 2, main manuscript). The concentration boundary around the middle body region will be dominated by the respiratory rate of the local reproductive system, minimizing the effect of the edges of the animal (head and tail), on the UME response. Similarly, in the head region measurements, the UME probes only the O_2 concentration boundary layer at edge of the animal and is not impacted by respiration of the entire body. In reality, the UME probes the local O_2 concentration over an area of the animal imminently close to it with regions further away impacting less its response. As OCR is dependent on the surface area and the 2D spherical model capture partially the nematode real geometry, the 2D axis-symmetric model is a reasonable approximation and adopted here for quantitative local OCR measurements.

S-6 UME as a local oxygen concentration probe

As the UME diffusion layer extends only to about 10 radii from the electrode, the O_2 reduction current recorded at the electrode is proportional to the average O_2 concentration at the edge of this concentration boundary layer, represented by the dashed line in Figure S7a.[13] As an approximation, the UME current can be seen as to be proportional to the O_2 concentration value at a point at the center of the UME disk, but 10 radii away (green semi-circle, Figure S7a). As the O_2 concentration boundary layer around the worm extends several microns into the solution (Figure S7b and Figure 3c of the main manuscript), the UME can be seen as a probe for the local oxygen concentration. Thus, the UME normalized current ratio can be calculated as the ratio between the O_2 concentration at the electrode height (20 and 40 μm) in the substrate boundary layer in a simulation only considering the substrate geometry (Figure S7b) divided by bulk concentration values.

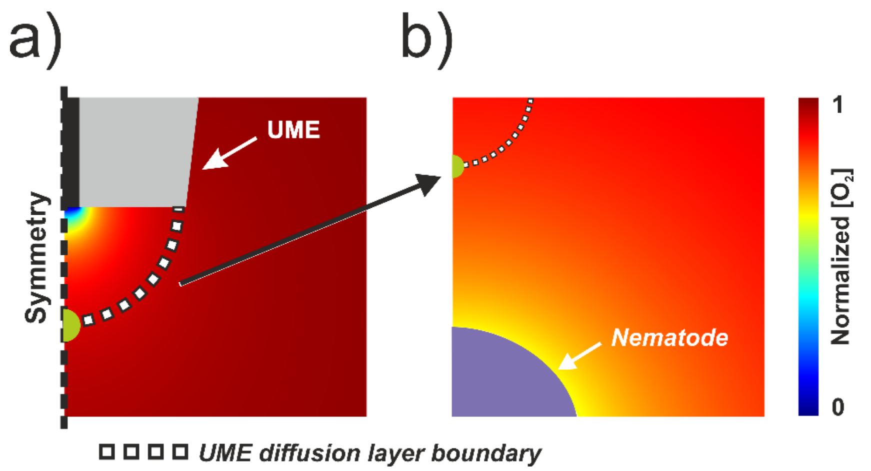

Figure S7: *O_2 concentration profile at (a) a 1.5 μm radius UME reducing oxygen at diffusion-limited conditions and (b) a sphere representing a worm with a local OCR of 6 pmol L^{-1}. In both figures, the dashed line represents the edge of the UME diffusion layer (10 electrode radii) where the concentration can be averaged to calculate the electrode current. The green semi-circle represents a point at 10 radii from the electrode center where the concentration approximates to the average of the diffusion layer edge.*

For comparison, the local OCR measurement for a head region was simulated in the two cases: with and without considering the electrode geometry. The variation between the two approaches gave an error of < 15 %. This approximation can facilitate the simulation and analyses of turnover rates over substrates, as the only calculation needed is for the concentration distribution over the substrate diffusion layer, ignoring the electrode geometry and electrodic process, which can be complicated. Most substrates can be approximated to regular geometric forms, for most of which analytical or semi-analytical solutions for transport equations exist,[14,15] allowing these calculations to be performed in a simple manner, similarly to other approaches reported in the literature.[16–18] However, this simplified approach is only valid as long as the electrode geometry does not interfere with the concentration boundary layer created by the sample, which can happen if the electrode, for instance, has a large insulating glass sheet around the electroactive area that can hinder oxygen transport from solution to the animal's surface when in close proximity to the sample. To model this interaction between the UME and the substrate diffusion layer, a full model, considering

the substrate and UME geometry (see S-4), is needed. This provides a complete insight in the electrode/substrate interaction, not possible in the simplified approach.

References

[1] M. Nebel, T. Erichsen, W. Schuhmann, Constant-distance mode SECM as a tool to Visualize local electrocatalytic activity of oxygen reduction catalysts, Beilstein J. Nanotechnol. 5 (2014) 141–151. doi:10.3762/bjnano.5.14.

[2] Y. Saito, A Theoretical Study on the Diffusion Current at the Stationary Electrodes of Circular and Narrow Band Types, Rev. Polarogr. 15 (1968) 177–187. doi:10.5189/revpolarography.15.177.

[3] J.F. Edmondson, G.N. Meloni, G. Costantini, P.R. Unwin, Synchronous Electrical Conductance- and Electron Tunnelling-Scanning Electrochemical Microscopy Measurements, ChemElectroChem. 7 (2020) 697–706. doi:10.1002/celc.201901721.

[4] J. Kwak, A.J. Bard, Scanning electrochemical microscopy. Theory of the feedback mode, Anal. Chem. 61 (1989) 1221–1227. doi:10.1021/ac00186a009.

[5] C.S. Santos, A.J. Kowaltowski, M. Bertotti, Single Cell Oxygen Mapping (SCOM) by Scanning Electrochemical Microscopy Uncovers Heterogeneous Intracellular Oxygen Consumption, Sci. Rep. 7 (2017) 11428. doi:10.1038/s41598-017-11956-w.

[6] D. Perry, D. Momotenko, R.A. Lazenby, M. Kang, P.R. Unwin, Characterization of Nanopipettes, Anal. Chem. 88 (2016) 5523–5530. doi:10.1021/acs.analchem.6b01095.

[7] G.N. Meloni, M. Bertotti, 3D printing scanning electron microscopy sample holders: A quick and cost effective alternative for custom holder fabrication, PLoS One. 12 (2017) e0182000. doi:10.1371/journal.pone.0182000.

[8] A.J. Bard, F.R.F. Fan, J. Kwak, O. Lev, Scanning electrochemical microscopy. Introduction and principles, Anal. Chem. 61 (1989) 132–138. doi:10.1021/ac00177a011.

[9] B. Erriah, S.J. Elliott, Experimental evidence for the role of paramagnetic oxygen concentration on the decay of long-lived nuclear spin order, RSC Adv. 9 (2019) 23418–23424. doi:10.1039/c9ra03748a.

[10] P. Han, D.M. Bartels, Temperature Dependence of Oxygen Diffusion in H 2 O and D 2 O †, J. Phys. Chem. 100 (1996) 5597–5602. doi:10.1021/jp952903y.

[11] R. Astrauskas, F. Ivanauskas, I. Morkvenaite-Vilkonciene, A. Ramanavicius, Mathematical Modelling of the Influence of Ultra-micro Electrode Geometry on

Approach Curves Registered by Scanning Electrochemical Microscopy, Electroanalysis. 31 (2019) 2214–2223. doi:10.1002/elan.201900313.

[12] Q. Dong, S. Santhanagopalan, R.E. White, Simulation of the Oxygen Reduction Reaction at an RDE in 0.5 M H[sub 2]SO[sub 4] Including an Adsorption Mechanism, J. Electrochem. Soc. 154 (2007) A888. doi:10.1149/1.2756994.

[13] C.G. Zoski, M. V. Mirkin, Steady-State Limiting Currents at Finite Conical Microelectrodes, Anal. Chem. 74 (2002) 1986–1992. doi:10.1021/ac015669i.

[14] D.D. Macdonald, The Mathematics of Diffusion, in: Transient Tech. Electrochem., Springer US, Boston, MA, 1977: pp. 47–67. doi:10.1007/978-1-4613-4145-1_3.

[15] A. Molina, J. Gonzalez, E.O. Barnes, R.G. Compton, Simple Analytical Equations for the Current–Potential Curves at Microelectrodes: A Universal Approach, J. Phys. Chem. C. 118 (2014) 346–356. doi:10.1021/jp409167m.

[16] T. Kaya, D. Numai, K. Nagamine, S. Aoyagi, H. Shiku, T. Matsue, Respiration activity of Escherichia coli entrapped in a cone-shaped microwell and cylindrical micropore monitored by scanning electrochemical microscopy (SECM), Analyst. 129 (2004) 529. doi:10.1039/b316582e.

[17] H. Shiku, T. Yasukawa, T. Matsue, T. Ito-Sasaki, M. Yokoo, H. Abe, S. Aoyagi, Oxygen Consumption of Mammalian Embryos and Oocytes Monitored by Scanning Electrochemical Microscopy, in: 2007 IEEE Sensors, IEEE, 2007: pp. 1408–1411. doi:10.1109/ICSENS.2007.4388676.

[18] R. Mukomoto, Y. Nashimoto, T. Terai, T. Imaizumi, K. Hiramoto, K. Ino, R. Yokokawa, T. Miura, H. Shiku, Oxygen consumption rate of tumour spheroids during necrotic-like core formation, Analyst. 145 (2020) 6342–6348. doi:10.1039/D0AN00979B.
